# Single cell analysis of short-term dry eye induced changes in cornea immune cell populations

**DOI:** 10.3389/fmed.2024.1362336

**Published:** 2024-03-15

**Authors:** Jehan Alam, Ebru Yaman, Gerda Cristal Villalba Silva, Rui Chen, Cintia S. de Paiva, Mary Ann Stepp, Stephen C. Pflugfelder

**Affiliations:** ^1^Ocular Surface Center, Department of Ophthalmology, Baylor College of Medicine, Houston, TX, United States; ^2^Human Genome Sequencing Center, Department of Molecular and Human Genetics, Baylor College of Medicine, Houston, TX, United States; ^3^Departments of Anatomy, Regenerative Biology and Ophthalmology, The George Washington University Medical School and Health Sciences, Washington, DC, United States

**Keywords:** dry eye, macrophage, cornea nerve, innate inflammation, CXCL1

## Abstract

**Background:**

Dry eye causes corneal inflammation, epitheliopathy and sensorineural changes. This study evaluates the hypothesis that dry eye alters the percentages and transcriptional profiles of immune cell populations in the cornea.

**Methods:**

Desiccating stress (DS) induced dry eye was created by pharmacologic suppression of tear secretion and exposure to drafty low humidity environment. Expression profiling of corneal immune cells was performed by single-cell RNA sequencing (scRNA-seq). Cell differentiation trajectories and cell fate were modeled through RNA velocity analysis. Confocal microscopy was used to immunodetect corneal immune cells. Irritation response to topical neurostimulants was assessed.

**Results:**

Twelve corneal immune cell populations based on their transcriptional profiles were identified at baseline and consist of monocytes, resident (rMP) and MMP12/13 high macrophages, dendritic cells (cDC2), neutrophils, mast cells, pre T/B cells, and innate (γDT, ILC2, NK) and conventional T and B lymphocytes. T cells and resident macrophages (rMP) were the largest populations in the normal cornea comprising 18.6 and 18.2 percent, respectively. rMP increased to 55.2% of cells after 5 days of DS. Significant changes in expression of 1,365 genes (adj *p* < 0.0001) were noted in rMP with increases in cytokines and chemokines (*Tnf, Cxcl1, Ccl12, Il1rn*), inflammatory markers (*Vcam, Adam17, Junb*), the TAM receptor (*Mertk*), and decreases in complement and MHCII genes. A differentiation trajectory from monocytes to terminal state rMP was found. Phagocytosis, C-type lectin receptor signaling, NF-kappa B signaling and Toll-like receptor signaling were among the pathways with enhanced activity in these cells. The percentage of MRC1^+^ rMPs increased in the cornea and they were observed in the basal epithelium adjacent to epithelial nerve plexus. Concentration of the chemokine CXCL1 increased in the cornea and it heightened irritation/pain responses to topically applied hypertonic saline.

**Conclusion:**

These findings indicate that DS recruits monocytes that differentiate to macrophages with increased expression of inflammation associated genes. The proximity of these macrophages to cornea nerves and their expression of neurosensitizers suggests they contribute to the corneal sensorineural changes in dry eye.

## 1 Introduction

The cornea is an avascular transparent tissue that serves as a principal barrier of the eye to mechanical, chemical, desiccating and microbial environment stresses (1, 2). The cornea’s unique structure, biochemical and immunological features contribute to the maintenance of corneal transparency (1, 3). The cornea is recognized to be an immune privileged tissue because of its avascularity, lack of mature antigen presenting cells, and tolerance related to anterior chamber-associated immune deviation (3–6). Immune privilege status is vital for an eye’s functional competence that when undermined by excessive inflammation causes corneal blindness (3). The cornea is home to a heterogenous immune cell population forming a complex immune network that responds to protect against many environmental damages and invasion of pathogenic microorganisms (1). Several immune cells type has been detected in the cornea, including neutrophils, dendritic cells, macrophages, natural killer cells, T cells, and innate lymphoid cells by flow cytometry or immunostaining using antibodies to conventional lineage markers (1, 3). Because antigens detected by these antibodies may be expressed by multiple cell types they may lack specificity to define certain cell populations (7). For example, the integrin CD11b was used as a marker to characterize macrophages/monocytes populations in the deep cornea stroma (5); however, CD11b is expressed by a variety of cells, including neutrophils, eosinophils, monocytes, macrophages, dendritic cells, and NK cells (7). Thus, the functional roles of the heterogenous CD11b^+^ immune cell population in causing inflammation or maintaining immune homeostasis in the cornea cannot be fully elucidated (1).

Single cell RNA sequencing (scRNAseq) is a powerful tool to reveal cellular diversity of a complex sample, with direct application to discover novel cell types and subtypes based on their gene expression profiles. Most of the scRNAseq studies in the cornea have focused on limbal stem cells, nonmyelinated Schwann cells, limbal region immune cells, or only myeloid cells (CD45^+^CD3^−^CD19^−^Ly6G^−^) sorted from the cornea (8–11). To date, a complete molecular characterization of CD45^+^ sorted immune cells in the cornea at the single cell level is lacking, and there are still many questions to be answered regarding which cornea immune cells produce regulatory or inflammatory factors in homeostasis and disease.

Dry eye is a prevalent condition that causes tear instability, corneal epithelial disease, and neurosensory perturbations. Inflammation has been implicated in the pathogenesis of the corneal epithelial disease and nerve sensitization that develops in dry eye. Increased levels of inflammatory mediators in tears, increased expression of inflammatory mediators by the corneal epithelium and recruitment of immune cells to the cornea has been detected in dry eye patients and/or mouse dry eye models. Previously reported studies have found an increase in CD11b^+^ cells detected by immunohistology and flow cytometry in mouse dry eye models (12). In this study, scRNA-seq was used to identify changes in corneal immune cell populations and their transcriptional profiles in an unbiased fashion.

## 2 Methods

### 2.1 Animals

The animal protocol for this study was designed according to the ARVO Statement for the use of Animals in Ophthalmic and Vision Research and was approved by the Institutional Animal Care and Use Committee at Baylor College of Medicine (Protocol AN-2032). Female C57BL/6 J (B6) (*n* = 50) mice aged 8–10 weeks were purchased from Jackson Laboratories (Bar Harbor, ME), and housed in a non-stressed (NS) environment at 50–75% relative humidity before the experiment.

### 2.2 Desiccating stress model of dry eye

Desiccating stress was induced by inhibiting tear secretion with scopolamine hydrobromide (Greenpark, Houston) in drinking water (0.5 mg/mL) and housing in a cage with a perforated plastic screen on one side to allow airflow from a fan placed 6 inches in front of it for 16 h/day for 5 days (DS5) as previously reported (13). Cages were placed in an environmentally controlled cabinet (Darwin Chamber, St. Louis, MO) with temperature of 75°F and relative humidity (RH) of 25%. Control mice were maintained in a non-stressed (NS) environmental cabinet maintained at 50–70% RH without exposure to an air draft.

### 2.3 Cell sorting

Adult mouse corneas anterior to the limbus were excised and incubated in 20 mM EDTA-PBS for 20 min, a total of 50 corneas from 25 mice were pooled and chopped with scissors into tiny pieces and incubated with 0.1% type IV Collagenase for 1 h to yield single-cell suspensions. The pooled samples were incubated with anti-CD16/32 (2.4G2, Catalog no. 553141, BD Pharmingen™, San Diego, CA), for 5 min at room temperature and subsequently stained with anti-CD45 (clone 30-F11, Catalog no. 103138, BioLegend) and with an infra-red fluorescent viability dye (Catalog no. L10119, Life Technologies, Grand Island, NY). The gating strategy was as follows: lymphocytes were identified by forward-scatter area (FSC-A) and side scatter area (SSC-A) gates, followed by two singlets gates (FSC-A vs. FSC-W and SSC-A vs. SSC-W) followed by live/dead identification using the infra-red fluorescent viability dye. The CD45^+^cells were sorted using the Aria-II cell sorter at the Baylor College of Medicine cytometry and cell sorting core.

### 2.4 Library preparation

Single-cell gene expression libraries was prepared using the Chromium Single Cell Gene Expression 3v3.1 kit (×10 Genomics) at the Single Cell Genomics Core at Baylor College of Medicine. In brief, single cells, reverse transcription (RT) reagents, Gel Beads containing barcoded oligonucleotides, and oil were loaded on a Chromium controller (×10 Genomics) to generate single-cell Gel Beads-In-Emulsions (GEMs) where full-length cDNA was synthesized and barcoded for each single cell. Subsequently the GEMs are broken and cDNA from every single cell is pooled. Following cleanup using Dynabeads MyOne Silane Beads, cDNA is amplified by PCR. The amplified product is fragmented to optimal size before end-repair, A-tailing, and adaptor ligation. The final library was generated by amplification.

### 2.5 Sequencing of 10X GEM 3’v3.1 single cell libraries

The BCM Genomic and RNA Profiling (GARP) Core initially conducted sample quality checks using the NanoDrop spectrophotometer and Agilent Bioana-lyzer 2,100. To quantitate the adapter-ligated library and confirm successful P5 and P7 adapter incorporations, the Applied Biosystems ViiA7 Real-Time PCR System and a KAPA Illumina/Universal Library Quantification Kit (p/n KK4824) was used. The GARP core sequenced the libraries on the NovaSeq 6,000 Sequencing System using the S2 v1.0 Flowcell as follows. Cluster Generation by Exclusion Amplification (ExAMP): Using the concentration from the ViiA7 TM qPCR machine above, 150 pM of the equimolar pooled library was loaded onto one lane of the NovaSeq S2 v1.0 flowcell (Illumina p/n 20,012,860) following the XP Workflow protocol (Illumina kit p/n 20,021,664) and amplified by exclusion amplification onto a nanowell-designed, patterned flowcell using the Illumina NovaSeq 6,000 sequencing instrument. PhiX Control v3 adapter-ligated library (Illumina p/n FC-110-3001) was spiked-in at 1% by weight to ensure balanced diversity and to monitor clustering and sequencing performance. The libraries were sequenced according to the 10X Genomics protocol, 28 cycles for Reads 1, 10 cycles each for the i7 and i5 reads, and 90 cycles for Read 2. An average of 251 million read pairs per sample was sequenced. FastQ file generation was executed using bcl2fastq and QC reports were generated using CellRanger v5.0.1 by the BCM Multiomics Core.

### 2.6 Bioinformatic analysis of scRNA-seq data

Raw sequence reads in the FASTQ format were aligned to the mouse reference genome using Cell Ranger Count v7.0.1 pipeline^1^ with the default settings for alignment, barcode assignment, and UMI counting of the raw sequencing data with genome reference Mouse (mm10) 2020-A. The resulting gene expression matrix was subjected to standardized quality control (QC) for single-cell RNA-Seq (scRNA) data to obtain clean feature count matrices from Cell Ranger outputs using Seurat.

### 2.7 Clustering, visualization, and cell annotation

Using Seurat_4.2.0 analysis package, first, we used the “FindVariableFeatures” function using “vst” method to identify a set of 2,500 genes that are highly variable that were used for downstream analysis such as dimensionality reduction and clustering. We then performed Principal Components Analysis (PCA) to construct a linear dimensionality reduction of the dataset that contain most of the complexity of the dataset. The cells were clustered in a graph-based approach within PCA space, and then non-linear dimensionality reductions were applied using t-distributed stochastic neighbor embedding (tSNE) and Uniform Manifold Approximation and Projection (UMAP) for further visualization purposes. Finally, differential gene expression (DEG) was performed using the “FindAllMarkers” function in Seurat to find cluster specific marker genes. Based on clusters specific DEG markers each cluster identity was predicted using the Cluster Identity Predictor (CIPR) web-based tool.^2^

### 2.8 Cell fate trajectory and pathway analyses

Trajectory analysis was performed with R package Monocle 3 ordering the cells in pseudotime along a trajectory.^3^ The data was pre-processed with clustering and dimensionality was reduced. A principal graph within each partition was fit using the learngraph function, and the results were visualized using the UMAP method. Subsequently, the cells were ordered according to their progress through the developmental program.

Sequencing data was analyzed for enriched KEGG (Kyoto Encyclopedia of Genes and Genomes) pathways in R as described (14). DEGs between non stressed and DS5 groups for each cell type were used to identify activated pathways.

### 2.9 Immunofluorescent staining and confocal microscopy

Freshly excised whole eyeballs were incubated in a paraformaldehyde-containing fixative as previously described (15) for 1 h and 15 min at 4°C, followed by two 15 min washes in 1X PBS + 0.02% NP40 with gentle shaking at room temperature (RT). The tissues were then incubated with pre-cooled Methanol:DMSO mixture at the ratio of 4:1 for 2 h at-20°C followed by transfer to 100% methanol at-20°C, tissues can be stored at this condition for several weeks. The tissue was permeabilized by incubating it in a 0.5% Triton-X-PBS wash buffer with a gradually decreasing methanol concentration (75, 50, 25, 0%) for 15 min each, with gentle shaking. Corneas were blocked with 2% FBS diluted in 1xPBS for 2 h at RT, followed by overnight incubation with anti βIII tubulin (1:100 dilution, Abcam) and Alexa-fluor 633 conjugated anti-MRC1 (1:100 dilution, BD Biosciences) antibodies. Next day, tissues were washed with 0.02% Tween-20 in 1xPBS 3 times for 30 min each with gentle shaking at RT, followed by incubation with Alexa-fluor 594 conjugated goat anti-rabbit IgG (1:200) diluted in blocking buffer for 2 h with gentle shaking at RT and then washed 0.02% Tween-20 in 1xPBS 3 times for 30 min with gentle shaking at RT. The samples were washed 3 times for 10 min with 1xPBS with gentle shaking at RT, mounted on slides, and flattened with coverslips. Immunostaining was visualized with a Nikon laser scanning confocal microscope (Nikon A1R MP, Nikon, Melville, NY, United States) at 0.9 μm Z-steps. The captured images were processed using NIS Elements Advanced Research (AR) software version 5.30.05 (Nikon).

### 2.10 Single-cell suspension and flow cytometry analysis

Adult mouse corneas anterior to the limbus were excised and incubated in 20 mM EDTA-PBS for 20 min, followed by chopping with scissors into tiny pieces and incubated with 0.1% type IV Collagenase for 1 h to yield single-cell suspensions. The samples were then incubated with anti-CD16/32 (2.4G2, Catalog no. 553141, BD Pharmingen™, San Diego, CA), for 5 min at room temperature and subsequently stained with surface markers anti-CD45 (clone 30-F11, Catalog no. 103138, BioLegend), anti-CD11b antibody (Clone M1/70, Cat. No. 552850, BD Pharmingen™, SanDiego, CA), anti-MRC1 (Clone Y17-505, Catalog no. 568808, BD Pharmingen™, SanDiego, CA), and anti-Mer (Clone 108,928, Catalog no. FAB5912G, R&D bio-techne, Eugene, OR) antibodies. Additionally, live/dead discrimination staining was performed using LIVE/DEAD™ Fixable Near-IR dye (Catalog. no. L10119A, Thermo Fisher Scientific Inc.). The gating strategy was as follows: lymphocytes were identified by forward-scatter area (FSC-A) and side scatter area (SSC-A) gates, followed by two singlets gates (FSC-A vs. FSC-W and SSC-A vs. SSC-W) followed by live/dead identification using the infra-red fluorescent viability dye.

### 2.11 CXCL1/KC ELISA

Cornea tissues anterior to the limbus area from normal (NS) and mice exposed to 5 days desiccation stress (DS5) were dissected and lysed using Pierce RIPA (Catalog no. 89900, Thermo Fisher Scientific Inc) buffer supplemented with protease inhibitors. Tissues were homogenized with scissors in RIPA buffer, followed by 30 min shaking and 14,000 rpm centrifugation at 4°C to remove cellular debris. Protein concentration for each sample lysate was determined by Micro BCA™ Protein Assay Kit (Catalog no. 23235, Thermo Fisher Scientific Inc). ELISA Quantikine Mouse CXCL1/KC immunoassay kit (Catalog no. MKC00B-1, R&D Systems) was used according to the manufacturer’s instructions. Briefly, 100 μL of each sample (250 μg/mL protein), mouse KC standards, and mouse KC control samples were added to an ELISA plate pre-coated with polyclonal antibodies specific to mouse KC and incubated for 2 h. Following washes, a horseradish peroxidase (HRP)-conjugated detection antibody was added and incubated for 2 h. After further washes, a tetramethylbenzidine (TMB) substrate solution was added for 30 min, and the reaction was stopped using a stop solution. The optical density (OD) was measured at 450 nm using a microplate reader. A standard curve was generated using the provided standards, and sample concentrations were interpolated from the standard curve. A student *t*-test was performed to determine the significance between the two groups.

### 2.12 Functional assays

Mice are placed in a cylindrical restrainer (Thermofisher, United States) and transient receptor potential (TRP) channel agonists were instilled and blinking, wiping and palpebral aperture dimensions were recorded with a video camera (Aluvium 1800, Allied Vision, Barrington, NJ, United States) placed at a fixed distance from the animal using StreamPix software (Norpix, Montreal, Canada). Blink and wiping were manually counted in 20s video segments.

### 2.13 Statistical analysis

Based on normality, parametric student T or nonparametric Mann–Whitney U tests were performed for statistical comparisons with an alpha of 0.05 using GraphPad Prism 9.0 software (GraphPad Software, Inc., San Diego, CA, United States).

## 3 Results

### 3.1 Single-cell RNA sequencing reveals diverse immune cell populations in the mouse cornea that change with desiccating stress

**Figure 1:**
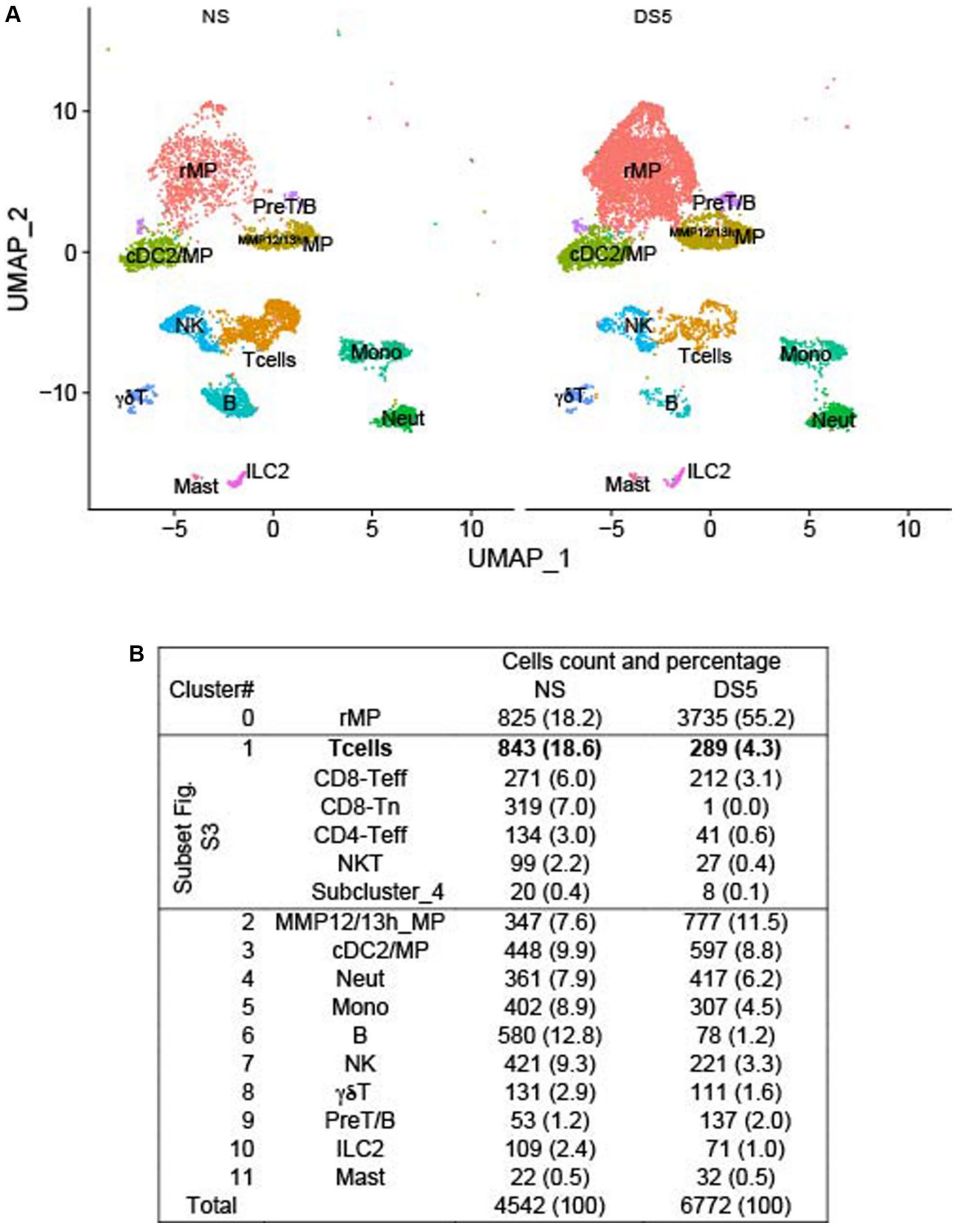
U map and table. Single-cell RNA sequencing. Single-cell RNA sequencing (scRNA-seq) revealed differences in cornea immune cell populations in C57BL/6 mice housed in nonstressed (NS) environmental conditions and after 5 days of desiccating environmental stress to induce dry eye (DS5). **(A)** UMAP of 12 distinct immune cell clusters generated from single-cell transcriptomic profiles of CD45^+^ cells sorted from the cornea using Seurat package V4.1.0. **(B)** Table of the cell count and percentage (parentheses) of the cells in each cluster. *n* = 25 mice (50 corneas) per group.

We employed droplet-based single-cell RNA sequencing (scRNA-seq) to evaluate the immune cell phenotype in the mouse cornea in an unbiased manner. We isolated CD45^+^ immune cells from the corneas of normal non-stressed (NS) and 5-day desiccating stress (DS5) exposed C57BL/6 J (B6) mice (*n* = 25, 50 corneas per group as biological replicates) and generated a transcriptomic profile of cells from NS and DS5 using the x10 Genomics platform. A modification of the standard DS dry eye model that substituted oral for subconjunctivally administered scopolamine was used (13). This model reduced tear volume to undetectable levels and caused corneal epithelial barrier disruption (Supplementary Figure S1). The resulting scRNA-seq data were passed through quality assessment filtering, standard pre-processing using Seurat_4.2.0 package (Supplementary Figure S2). After quality control we merged the two data sets and analyzed 4,542 cells from NS and 6,772 cells from DS5 with 4,000 variable features. We performed graph-based clustering and identified 12 distinct cell clusters (Figure 1A) based on the expression of signature marker genes listed in Supplementary Table S1. A dot-plot (Supplementary Figure S3) reveals the specificity and level of expression of signature marker genes in each cluster. The count and percentage of cells in each cluster in the entire NS and DS5 populations are shown in Figure 1B. The identified clusters included two types of macrophage (resident macrophages – rMP) and (MMP 12 and 13 high – ^MMP12/13^MP), type 2 conventional dendritic cells (cDC2)/macrophage, T cells (consisting of 5 subclusters shown in Supplementary Figure S4), neutrophils, monocytes, B cells, natural killer cell (NK), γδT cells, pre-T/B cells, innate lymphocyte type 2 (ILC2) and mast cells. Myeloid cells (rMP, ^MMP12/13^MP, mono, neutrophil and cDC2) comprise 52.5% of the total cell population, and the remaining 47.5% consist of lymphoid cells (B, Cd8, CD4T, NK, NKT, γδT, ILC2 and Pre-T/B). Most striking change in cell percentage following the induction of dry eye is an increase in rMP from 18.2 to 55.2%. The cornea has previously been found to have two populations of macrophages: CCR2^lo^rMP which may be there from embryogenesis and recruited CCR2^hi^MP that are recruited to the cornea in response to chemokines that signal through CCR2 (e.g., CCL2) that increase from inflammatory stimuli such as dry eye (16, 17). Overall, our single-cell transcriptomic atlas shows a diverse immune cell population in the cornea of B6 mice that is composed of cells with distinct gene expression profiles and a marked increase rMP.

### 3.2 Myeloid cell trajectory and differentially expressed genes

**Figure 2:**
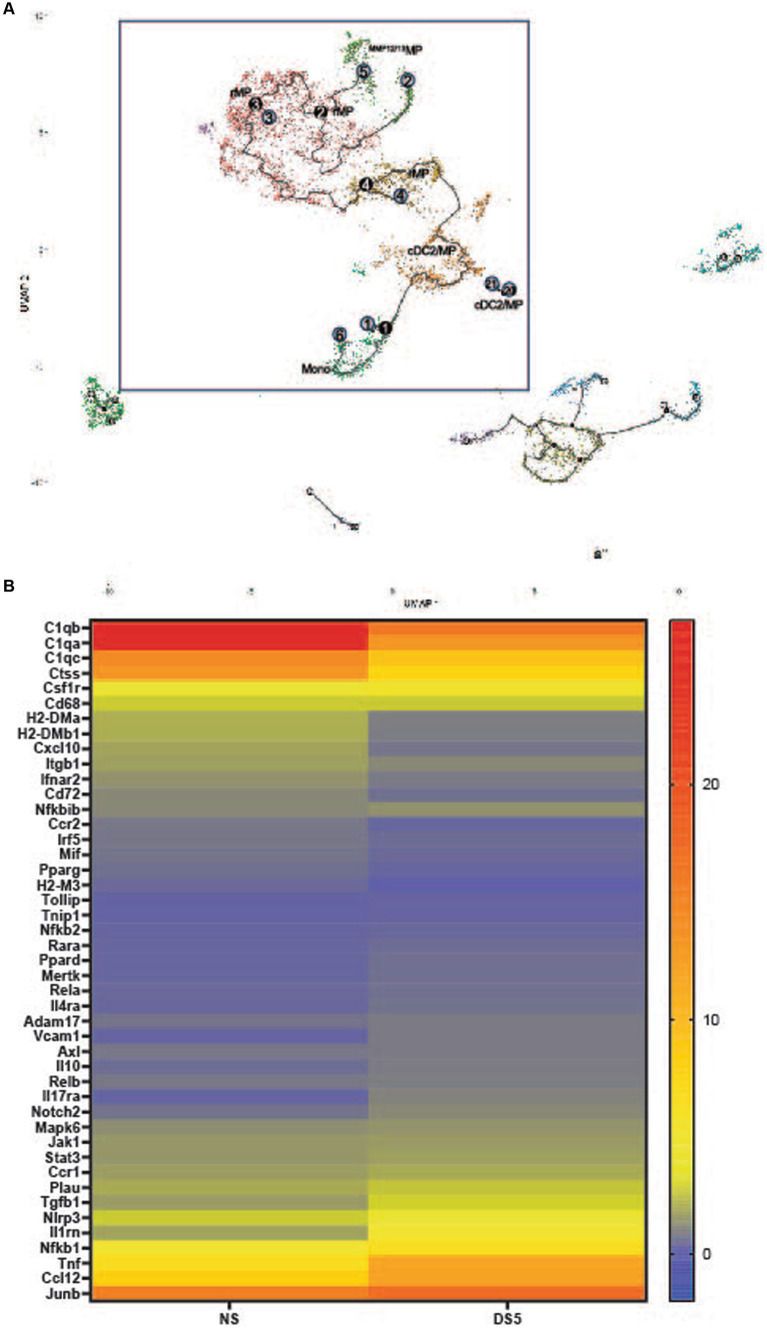
**(A)** Myeloid trajectory tree. Pseudotime with numbers showing stages of differentiation in the myeloid trajectory tree (black box). Black circles are branch nodes (splitting of the differentiation pathway), and light gray circles are differentiation outcomes. Cluster descriptions correspond to Seurat annotations. **(B)** Heat map of top differentially expressed genes in cluster 0. The top 45 differentially expressed genes (adj *p* value <0.0001) compared to the non-stressed group in Cluster 0 macrophages. Scale is log2 fold.

Cell trajectories based on transcriptional profiles were calculated using Monocle 3. The myeloid trajectory that includes the macrophage populations with the greatest increase in DS is shown in the box in Figure 2A. The numbers show the relative position in the trajectory with higher numbers indicating a later point in the trajectory than cells with lower numbers. Monocytes are the starting point in this trajectory that extends through cDC2/MP and rMP and branches and terminates in the ^MMP12/13^MP. This suggests that the increase in rMP results from differentiation of monocytes recruited from the blood. This is supported by low expression of the proliferation gene *Mki67* by rMP.

A heatmap of the top 50 differentially expressed genes (DEGs) in cluster 0 rMP is shown in Figure 2B. The gene expression profile shows changes in macrophage (Cd68, *Ccr2, Pparg, Junb* and MHCII associated), phagocytic (*C1qa-c*, *Il10*, *Tgfb*), TAM (Tyro, Axl, Mer) receptor (*Mertk, Axl*), protease (*Adam17, Ctss, Plau*), inflammation signaling genes (*Nfkb1* and *2*, *Rela* and *Relb*, *Jak1, Stat3, Mapk6*) and cytokine/chemokine (*Il1rn, Tnf, Ccl12, Il17ra*) genes. Most notable is the decrease in certain phagocyte associated genes (*C1qa-c, Pparg*) and increases in inflammatory (*Csf1r, Ccr1, Adam17, Nlrp3, Nfkb1, Tnf, Ccl2*) and anti-inflammatory (*Tgfb1, IL10, Il1rn*) genes. Features plots of cell associated gene expression in C0 rMP at DS5 (Supplementary Figure S5) show most cells express *C1qb*, *Junb*, the type 1 (M1) MP marker *Nos2, CD68, Nlrp3, Nfkb1* and *Tnf*, while few cells express the M2 marker *Arg1, Il12b* and the anti-inflammatory/homeostatic MP factor *Cd183* (*Cx3cr1*).

These findings indicate that dryness alters gene expression in the rMP cluster, in particular expression of innate inflammatory genes.

### 3.3 Pathway analysis

**Figure 3:**
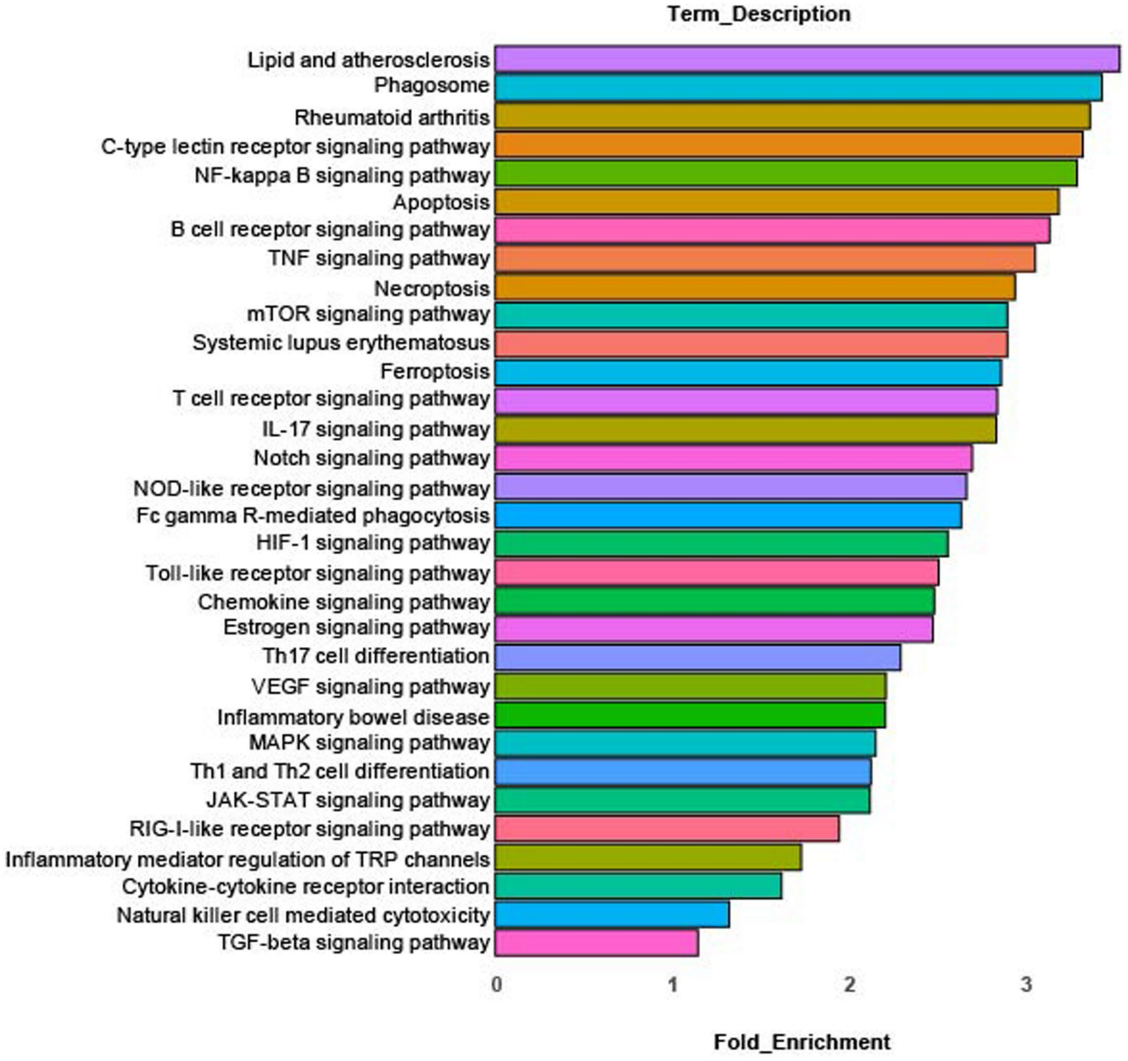
KEGG pathway analysis. Enriched canonical KEGG pathways in rMP cluster [rMP (NS) vs. rMP(DS5)] that show significant differences (adj *p* = 0.006) between NS and DS5.

KEGG pathway analysis of differentially expressed genes in cluster 0 identified the enriched pathways shown in Figure 3. These pathways include: innate inflammation, microbial product response, inflammatory signaling, cytokine and cytokine receptor signaling, autoimmune/inflammatory disease and inflammatory mediator regulation of TRP channels. Complete KEGG pathway data is found in Supplementary Table S2.

### 3.4 Immunolocalization of rMP

**Figure 4:**
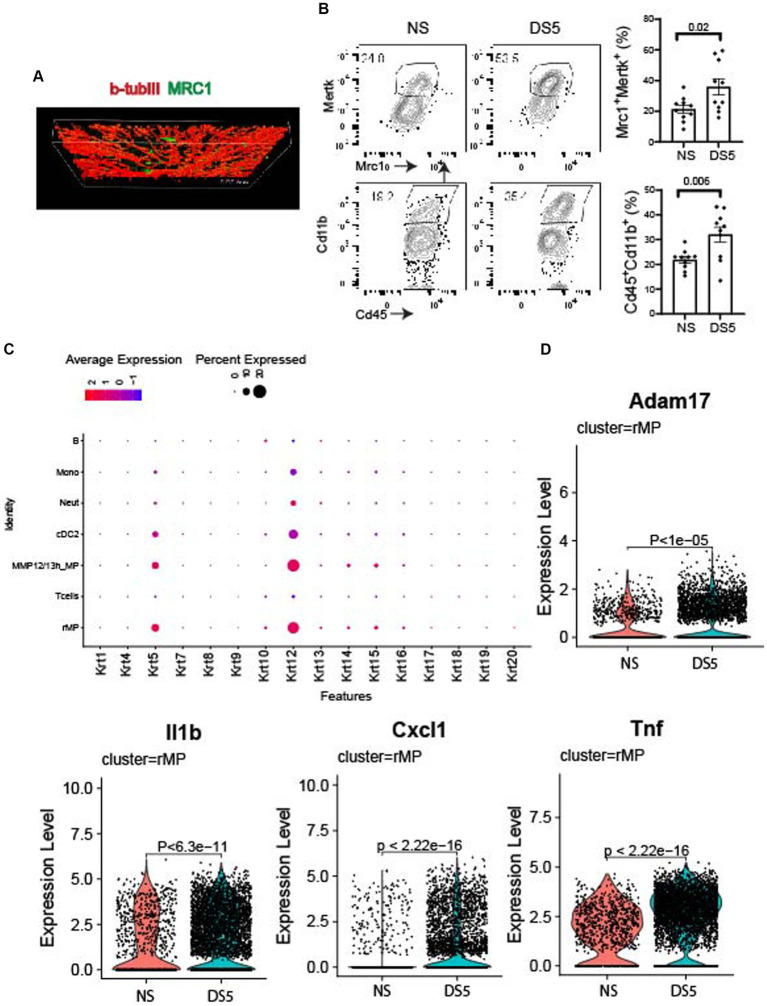
Immunolocalization and expression of phagocytic and neurosensitizing genes. **(A)** Confocal Z-stack image taken in the center of a cornea wholemount from the non-stressed (NS) group showing the basal epithelial plexus stained for cornea nerve marker BIII tubulin (βIIItub, red) and macrophage marker MRC1 (green). **(B)** Single cell suspensions prepared from corneas of C57BL/6 (B6) mice housed in normal humidity (NS) or exposed to desiccating stress for 5 (DS5) days were stained with anti-CD45, anti-CD11b, anti-MRC1 and anti-MERTK. Flow cytometry scatter plot shows CD11b^hi^ and MRC1/MERTK^hi^ cells; Bar graphs show mean percentages of cells in each group (*n* = 10). The error bars indicate the standard error of mean (SEM), Student *T*-test was used for statistical comparison. **(C)** Dot plot of cytokeratin genes in phagocytic (rMP, MMP12/13MP, cDC2, neutrophils, and monocytes) and non-phagocytic cells (T and B lymphocytes). **(D)** Violin plots of *Adam17*, and neurosensitizers (*Il1b, Cxcl1, Tnf*).

Mrc1 (CD206), is a rMP lineage defining cell surface c-type lectin that scavenges unwanted high mannose N-linked glycoproteins found on the surface of microbes, including bacteria, fungus, viruses and parasites (18). We used this antigen as a marker to detect these cells in the cornea. Compared to NS, an increased percentage of MRC1+ cells in the cornea was measured after 5 days of DS by flow cytometry (Figure 4A). MRC1^+^ cells are found in the corneal epithelium in proximity to basal epithelial nerve plexus that is stained for βIII tubulin (Figures 4A,B). These cells may have defensive roles during homeostasis in the unstressed cornea by phagocytosing pathogens, foreign antigens, and apoptotic cells. This function is supported by detection of cornea specific keratin 12 expression by these cells as well as other phagocytic cells in the myeloid trajectory. The dot plot of 16 different keratin (Krt) genes shows increased levels of *Krt12* in these cells that may be from phagocytosed apoptotic epithelial cells or exosomes from these cells (Figure 4C). We are unable to amplify *Krt12* from cultured BM derived monocytes (data not presented).

Increased expression of inflammatory mediators, such as Adam17 (Figure 4D), an inflammatory protease that cleaves numerous cell membrane cytokines and receptors, including the TAM receptor MERTK (19–22). Cleavage of MERTK results in reduced phagocytotic clearance of apoptotic cells and pro-inflammatory nucleic acids (19, 23, 24). We also found Increased expression of neurosensitizers such as *IL1b, Cxcl1,* and *Tnf* (25, 26). Significant changes in expression of other phagocytic, inflammatory, protease and anti-inflammatory genes are also seen in other cells in the myeloid trajectory: monocytes and ^MMP12/13^MP (Supplementary Figure S6) and cDC2 (Supplementary Figure S7).

### 3.5 CXCL1 is a neurosensitizing chemokine in dry eye

**Figure 5:**
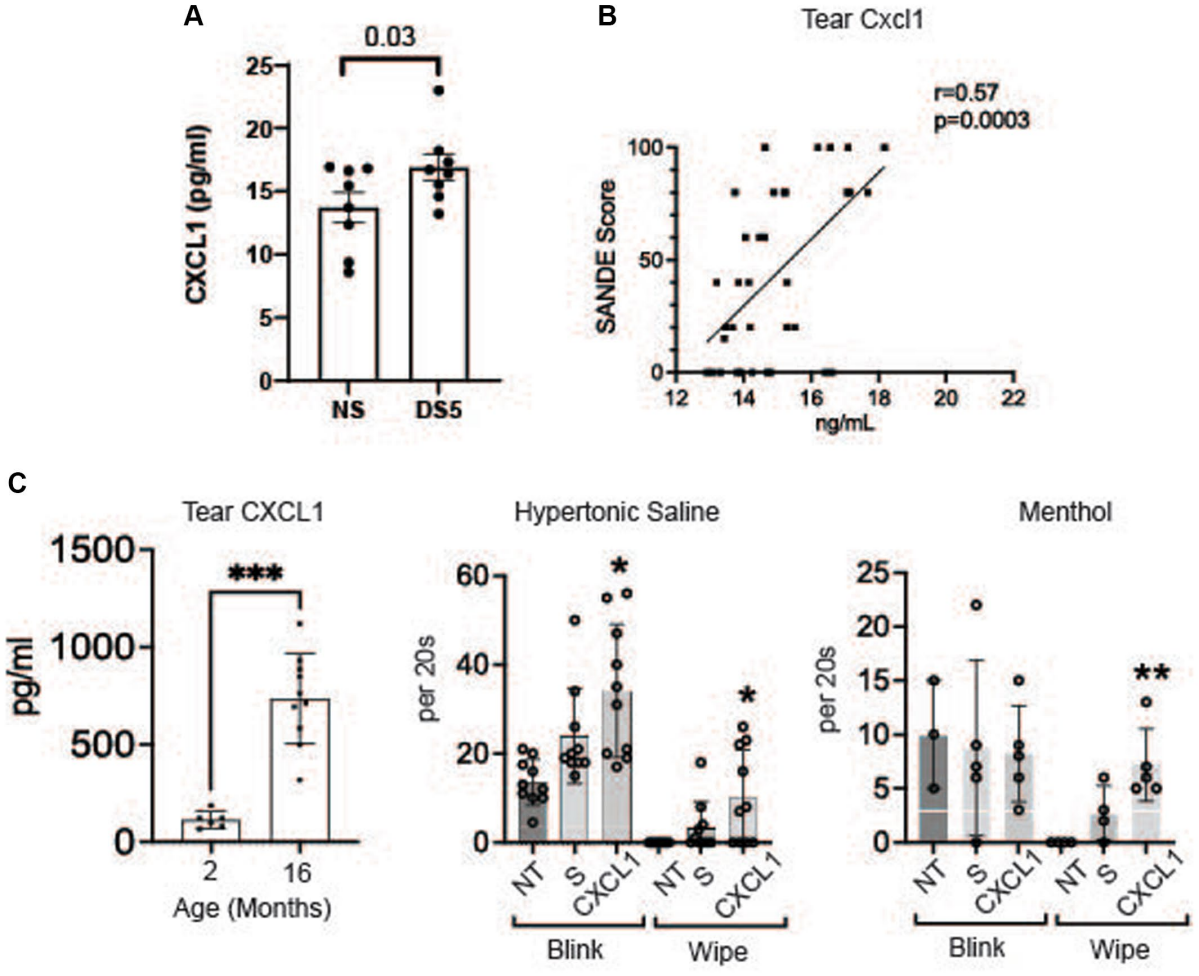
CXCL1 and corneal nociceptor sensitization. **(A)** Mean concentrations of CXCL1 in corneal lysates of NS and DS5 corneas detected by ELISA (*n* = 8). Student *T*-test was used for statistical comparison. **(B)** Correlation of SANDE eye discomfort questionnaire scores and tear CXCL1 concentration in 17 normal control subjects and 15 primary SS dry eye patients; Data is from a previously published study (27); **(C)** Tear concentration of CXCL1 in 2 M and 16 M old C57BL/6 mice measured by Luminex immunobead assay (28) (***p ≤ 0.001); **(D)** Functional markers of discomfort/pain (blink and wipe) are compared in C57BL/6 mice following instillation of TRPV1 agonist 5% hypertonic saline (HS), or TRPM8 agonist 0.2 μM menthol. 8-week-old B6 mice (*n* = 8) received no treatment (NT) or were treated topically with 2 μL of 0.9% saline (S) or CXCL1 4x on day-1 and 1x at 8:00 AM on day 0 and markers of discomfort/pain (blink and wipe) in response to topically applied HS or menthol (20 μM) were compared at 9:00 AM on day 0. CXCL1-treated mice had significantly greater blink and wipe response to HS and greater wiping to menthol than other groups (*p ≤ 0.01, **p ≤ 0.001).

The concentration of chemokine CXCL1 (GROα) in the cornea significantly increased after 5 days of DS (Figure 5A). CXCL1 (GROα) has been found to increase in the tears and conjunctiva of patients with SS KCS (27, 29). We found a correlation between tear CXCL1 concentration and severity of ocular irritation using data from our published survey of tear inflammatory mediators in SS (Figure 5B) (27). We have also found an increase in CXCL1 in the tears of aged mice that develop dry eye (Figure 5C) (28). Because CXCL1 is recognized to be a nociceptor sensitizer involved in chronic pain and TRPV1 activator (30, 31) we evaluated if topical application of CXCL1 to the ocular surface heightened the pain response to topically applied TRPV1 (hypertonic saline) and TRPM8 (menthol) agonists (32–35). We found CXCL1 increased both blink and wipe responses to hypertonic saline, but it only increased blink rate following menthol treatment (Figure 5D).

## 4 Discussion

Our study compared cornea immune cell populations using single cell RNA seq between mice maintained in normal environmental conditions and those with experimentally induced short-term dry eye. The cornea was found to contain 12 immune cell populations, that includes several types of monocytes, macrophages, neutrophils, dendritic cells, innate and conventional lymphocytes and mast cells. The greatest dry eye induced change in immune cell populations is a greater than 3-fold increase in macrophages designated as resident macrophages (rMP) based on their gene expression profile. In homeostatic conditions these cells may function to suppress inflammation by clearing microbes, foreign antigens, and dead cells. They express genes associated with phagocytosis and apoptotic cell clearance, including *C1q* complement factors, *Apoe, Mertk, Il10, Tgfb* and the mannose receptor *Mrc1* which we used as a marker to immunodetect these cells in the cornea (18, 36–39). The rMP along with other phagocytic cells (^mmp12/13hi^MP, cDC2/MP, neutrophils and monocytes) express the cornea specific *Krt12* gene that may be from engulfed apoptotic epithelial cells or exosomes released by epithelial cells. It remains to be determined if the observed change in macrophages is sustained in chronic dry eye.

The mouse cornea has been found to contain dendritic cells and macrophages. Hamrah and colleagues immunodetected CD11c^−^CD11b^+^ macrophages predominantly in the posterior cornea stroma and cells positive for the monocyte marker CD14+ throughout the stroma using confocal microscropy (40). Another study reported the presence of two macrophage populations in the mouse cornea, CCR2^lo^ cells that are present from embryogenesis and can proliferate *in vivo*, and CCR2^hi^ cells that are repopulated from monocytes recruited from the blood in steady state and following epithelial injury or during inflammation (16). CD11b^+^ cells were noted to increase in the cornea in a DS dry eye model like the one used in our study (41). Antibody neutralization of CCR2 was found to decrease the number of infiltrating CD11b^+^ cells, expression levels of IL-1α and-β in the cornea, and T cells in the conjunctiva (12).

Because CD11b (*Itgam*) is expressed by a variety of myeloid lineage immune cells, it lacks specificity as a marker to identify the specific cell types that infiltrate the cornea during inflammatory stresses such as dry eye (7). Furthermore, antigen characterization does not provide information about expression of pathogenic factors that can cause epithelial disease or irritation/pain. The unbiased single cell sequencing approach used in our studies identifies cells at a deeper level than immunological methods and provides relevant information about DS induced changes in expression of anti-and pro-inflammatory factors.

it is possible the rMPs increase to clear the increased corneal epithelial cell apoptosis that develops in this dry eye model (42, 43). Indeed, significantly increased expression of corneal keratins *Krt5* and *Krt12* was found in the ^mmp12/13hi^MP. Based on the modeled trajectory from monocytes to MPs, the decrease in percentage of monocytes accompanying the increase in macrophages in DS and the low expression of the proliferation gene *Mki67* it appears that the increase in number and percentage of MPs can be attributed to monocytes recruited from the blood. This is consistent with our previous report that DS stimulates CCR2 dependent monocyte recruitment and cascade to MP in the conjunctiva (17). CCR2 decreases with monocyte differentiation to macrophages and decreased CCR2 expression was noted at DS5 (17).

Macrophage differentiation is conditioned by the environment. Tissue resident macrophages are conditioned to function as phagocytes and maintain immune tolerance (44, 45). The dry eye environment with epithelial activation and increased concentrations of inflammatory mediators in the tears causes a shift in corneal MP gene expression with reduced expression of certain homeostatic factors and increased expression of inflammatory mediators. The rMP express genes associated with type 2 (M2) macrophages during non-stressed conditions and during DS. At DS5 expression of certain anti-inflammatory mediators/pain suppressors (i.e., *Il10 and Tgfb1*) increased, as did expression of inflammatory cytokines, chemokines and signaling pathway molecules (46, 47).

We found MRC1^+^ rMP are located in the basal epithelium in the vicinity of the corneal epithelial nerve plexus. Certain factors produced by these cells during homeostasis (such as IL-10 and TGF-β1) may regulate nerve tone and suppress pain inducing signaling in response to physiological insults to the cornea. Macrophages (CX3CR1^+^, IBA^+^, MHCII^+^, F4/80^+^, CD11b^+^) have been previously found to be associated with nerves in the peripheral corneal stroma. These cells dissociated from the nerves within 2 h following central epithelial debridement and returned to normal density by 72 h post injury (48). A subsequent study found 40–50% fewer immune cells in the peripheral corneal epithelium 3 h post-trephine injury compared to the number in unwounded corneas. All of the immune cells identified in the peripheral corneal epithelium were associated with intraepithelial corneal nerves (49). DS increased expression several nociceptor sensitizers, including CXCL1 which is a recognized TRPV1 channel sensitizer (31, 50, 51). CXCL1 has been found to increase in conjunctiva and tears in Sjögren syndrome dry eye, in tears of aged C57BL/6 mice and in the corneal epithelium of the CD25 knockout mouse Sjögren syndrome model (29, 52, 53). Topically applied CXCL1 heightened pain responses (blink and wiping) to topically hyperosmotic saline, a TRPV1 agonist. This is a relevant stimulus in dry eye, because tear osmolarity increases in dry eye and may reach levels as high as 900 mOsm/L in areas of precorneal tear breakup (54).

CXCL1 has also been found to activate Adam17 which can further amplify inflammation by cleaving cell membrane TNF and MERTK (19, 55). ADAM17 gene expression has also been found to be increased in SS (19, 55, 56). ADAM17 has been found to participate in other processes in the cornea. Adam 17 and heparin binding EGF (HB-EGF) were reported to mediate activation of the EGF receptor (EGFR) in corneal epithelial cells (57). MMP-9 and Elevated levels of ADAM17 and cleavage of the extracellular domain of α6β4 hemidesmosomal integrin by this and several other matrix metalloproteinases was associated with recurrent corneal erosions after corneal debridement wounding (58). Additionally, ADAM17 contributes to development of corneal erosions following exposure to sulfur mustard and inhibiting ADAM17 inhibits detachment of the from the stroma (59, 60).

In conclusion, this study found short term dry eye causes an increase in macrophages and a shift to greater expression of inflammatory and neurosensitizing genes. It provides evidence to perform additional studies to investigate the pathogenic role of macrophages in dry eye associated corneal disease and eye discomfort. A strength of this study is the use of scRNA-seq to identify changes in corneal immune cells and their expression patterns. A weakness is the effects of chronic dry eye on cornea immune cell populations and the effects of dry eye on other cornea cell populations (i.e., epithelial cells and keratocytes) was not evaluated.

## Data availability statement

The datasets presented in this study can be found in online repositories. The names of the repository/repositories and accession number(s) can be found at: https://singlecell.broadinstitute.org/single_cell/study/SCP2448/single-cell-analysis-of-desiccation-induced-changes-in-cornea-immune-cell-populations.

## Ethics statement

The studies involving humans were approved by Baylor College of Medicine IRB. The studies were conducted in accordance with the local legislation and institutional requirements. Written informed consent for participation was not required from the participants or the participants’ legal guardians/next of kin in accordance with the national legislation and institutional requirements. The animal study was approved by Baylor College of Medicine IACUC. The study was conducted in accordance with the local legislation and institutional requirements.

## Author contributions

JA: Writing – review & editing, Writing – original draft, Methodology, Investigation, Formal analysis, Data curation, Conceptualization. EY: Writing – review & editing, Writing – original draft, Methodology, Investigation, Formal analysis. GS: Writing – review & editing, Writing – original draft, Validation, Software, Methodology, Formal analysis, Data curation. RC: Writing – review & editing, Supervision, Methodology, Funding acquisition, Data curation, Conceptualization. CSP: Writing – review & editing, Supervision, Methodology, Investigation, Funding acquisition, Conceptualization. MS: Writing – review & editing, Validation, Methodology, Funding acquisition, Conceptualization. SP: Writing – review & editing, Writing – original draft, Methodology, Investigation, Funding acquisition, Formal analysis, Conceptualization.

## Footnotes

^1^ https://cloud.10xgenomics.com

^2^ https://aekiz.shinyapps.io/CIPR/

^3^ https://cole-trapnell-lab.github.io/monocle3
