# Erratum: Single cell analysis of short-term dry eye induced changes in cornea immune cell populations

**DOI:** 10.3389/fmed.2024.1414997

**Published:** 2024-04-25

**Authors:** Frontiers Production Office

**Affiliations:** Frontiers Media SA, Lausanne, Switzerland

**Keywords:** dry eye, macrophage, cornea nerve, innate inflammation, CXCL1

Due to a production error, an author name was incorrectly spelled as “Cinita S. de Paiva.” The correct spelling is “Cintia S. de Paiva.”

The publisher apologizes for this mistake. The original article has been updated.

