# Supplementary material for: Single cell analysis of short-term dry eye induced changes in cornea immune cell populations
Source: Front Med (Lausanne). 2024 Mar 15;11:1362336. doi: 10.3389/fmed.2024.1362336 (PMC10978656; doi:10.3389/fmed.2024.1362336)
Supplement: Supplementary file 1 [file Table_1.DOCX]

Supplementary Material

**
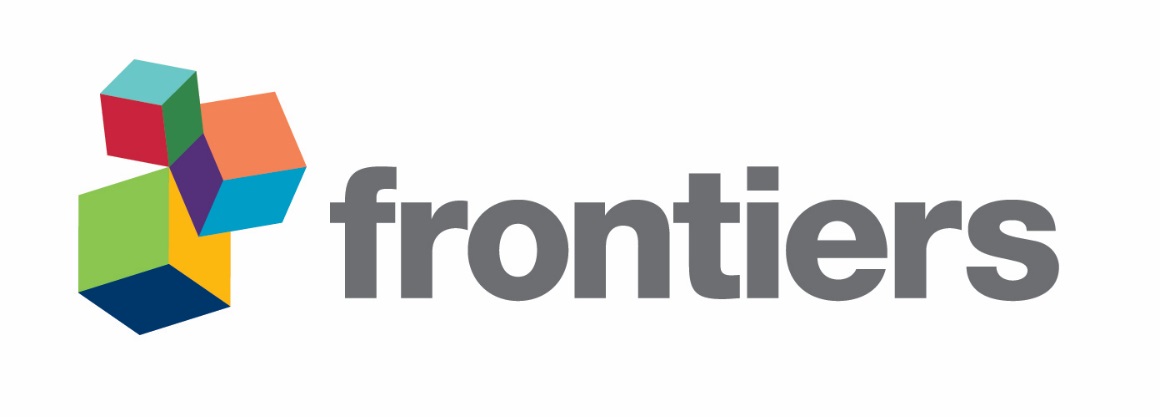
**

**Supplemental Figure Legends**

S1. QC A. Quality control for selecting cells for further analysis. Plots showing RNA counts (molecules), unique features (genes), and percent mitochondrial (mt) genomes detected per cell for all CD45^+^ cells in each experimental group (top) and in each cell cluster (bottom). B. Umap feature plots of cell lineage defining genes.

S2. Dot plot expression profiles of all cell clusters showing relative expression levels of gene expression. Size of the dot equates to the percent of cells in the cluster expressing the marker. Color of dots based on natural log of the normalized RNA expression.

S3. Dot plot expression profiles of T cell clusters showing relative expression levels of gene expression. Size of the dot equates to the percent of cells in the cluster expressing the marker. Color of dots based on natural log of the normalized RNA expression.

S4. Features plots of cell associated gene expression in C0 rMP at DS5 of top differentially expressed genes, and MP associated genes including the type 1 (M1) MP marker Nos2 and the M2 marker Arg1.

S5. Violin plots of genes that significantly increased at DS5 (PF_63067) compared to NS (PF_61353) in A. monocytes and B. ^MMP12/13^MP.

S6. Violin plots of genes that significantly increased at DS5 (PF_63067) compared to NS (PF_61353) in cDC2/MP.

**Supplementary Tables**

1. Signature marker genes identified with CIPR

2. KEGG pathways
