# Supplementary figures and images for: Single cell analysis of short-term dry eye induced changes in cornea immune cell populations
Source: Front Med (Lausanne). 2024 Mar 15;11:1362336. doi: 10.3389/fmed.2024.1362336 (PMC10978656; doi:10.3389/fmed.2024.1362336)

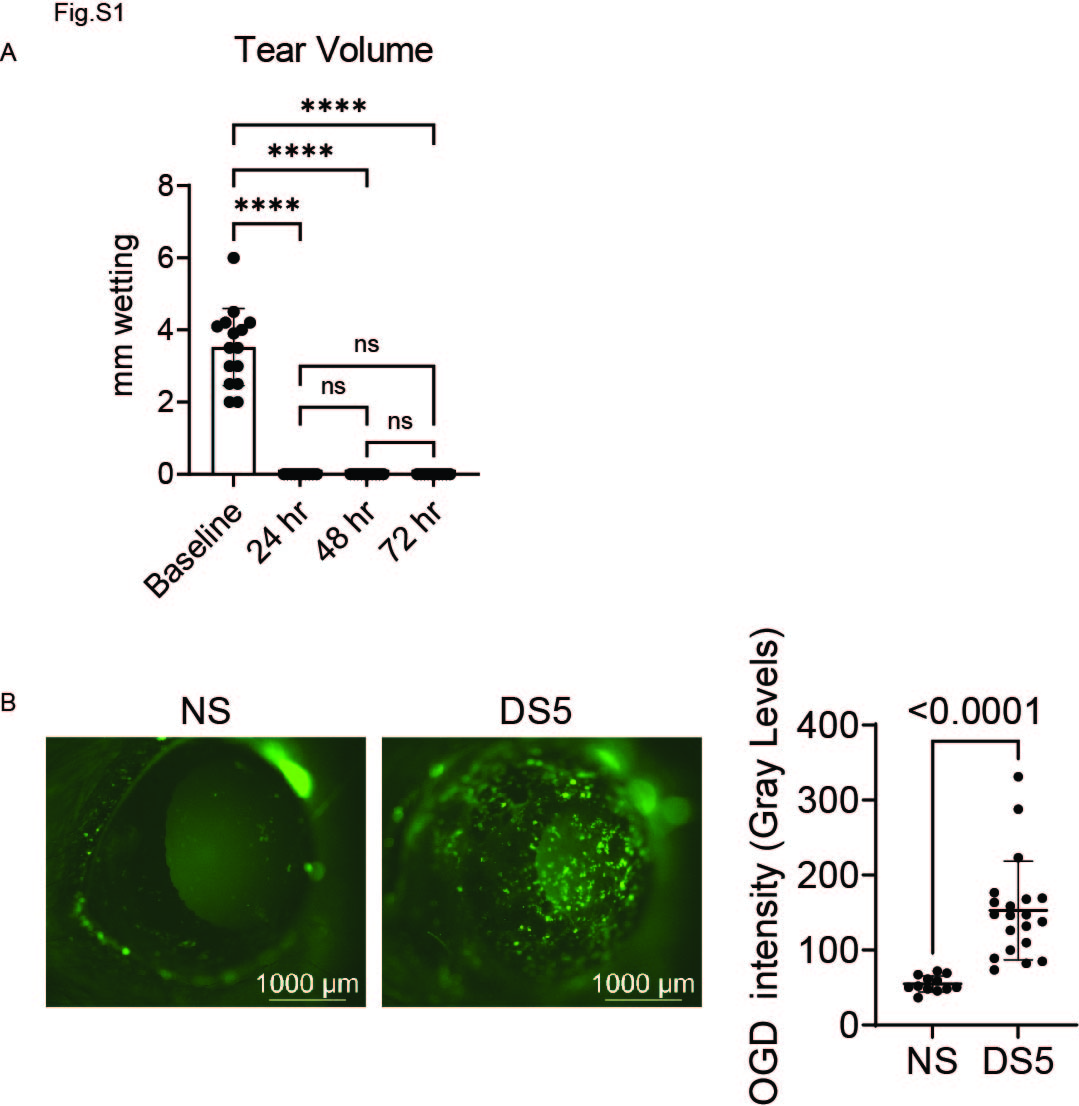

Supplement: Supplementary file 4 [file Image_1.jpg]

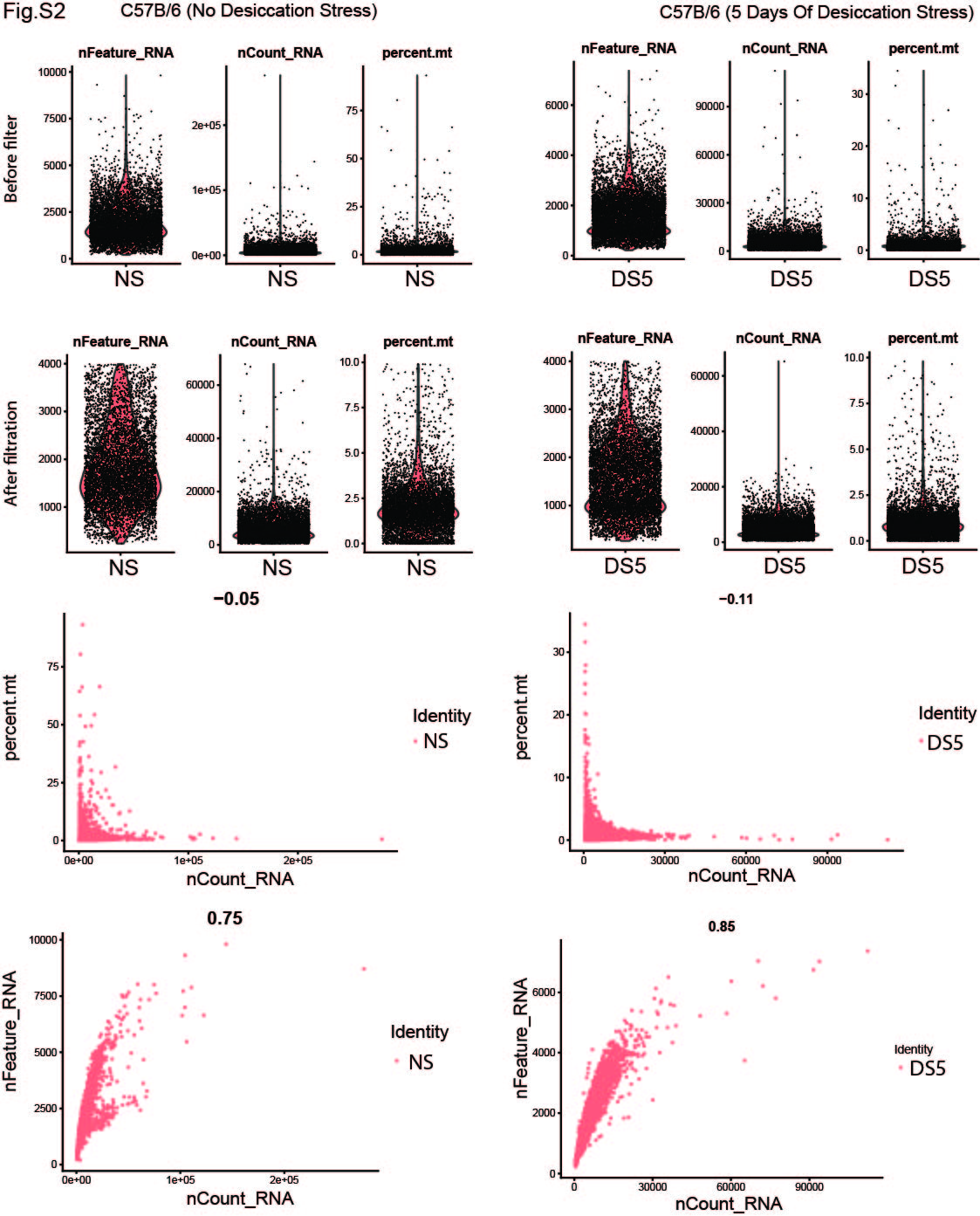

Supplement: Supplementary file 5 [file Image_2.jpg]

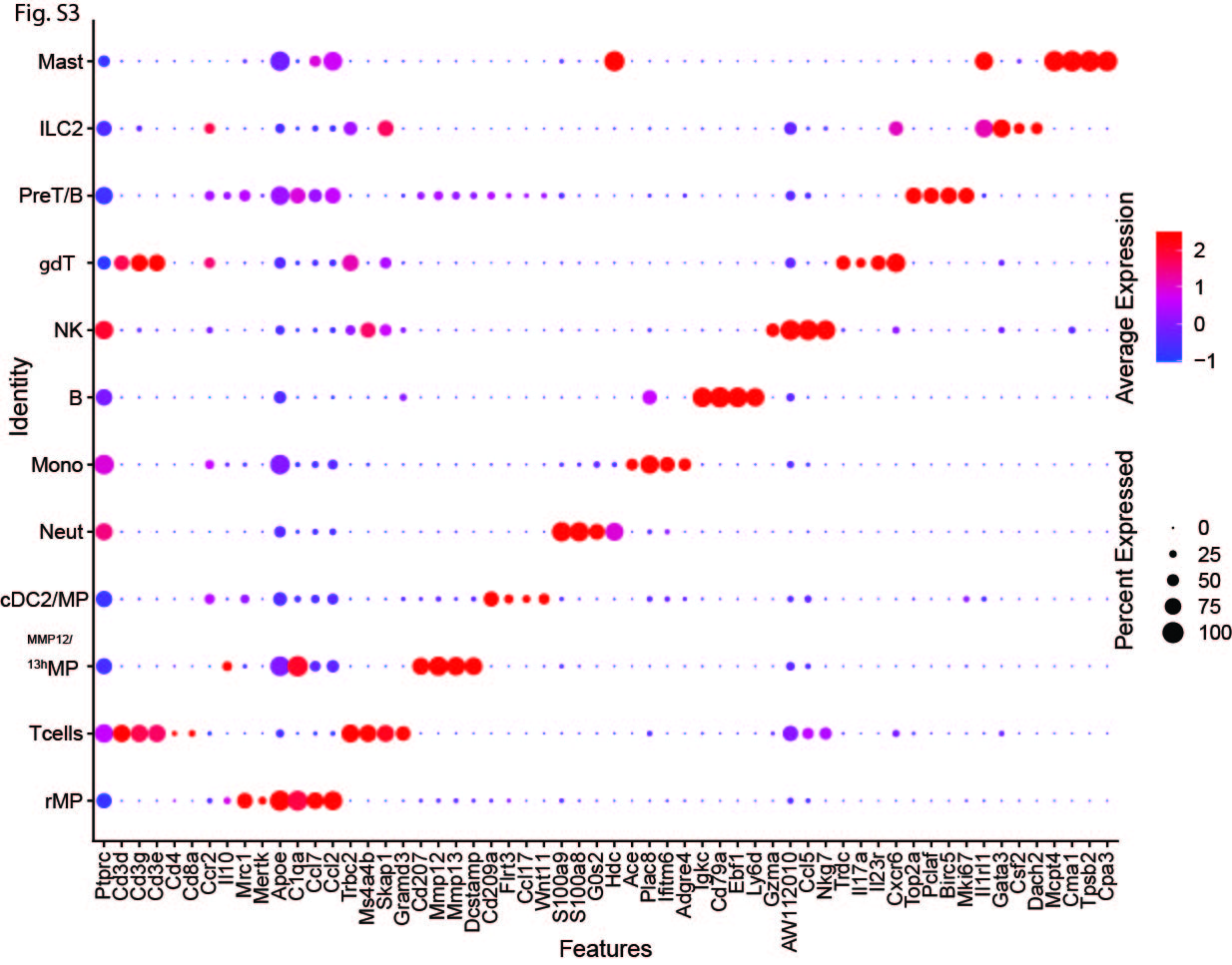

Supplement: Supplementary file 6 [file Image_3.jpg]

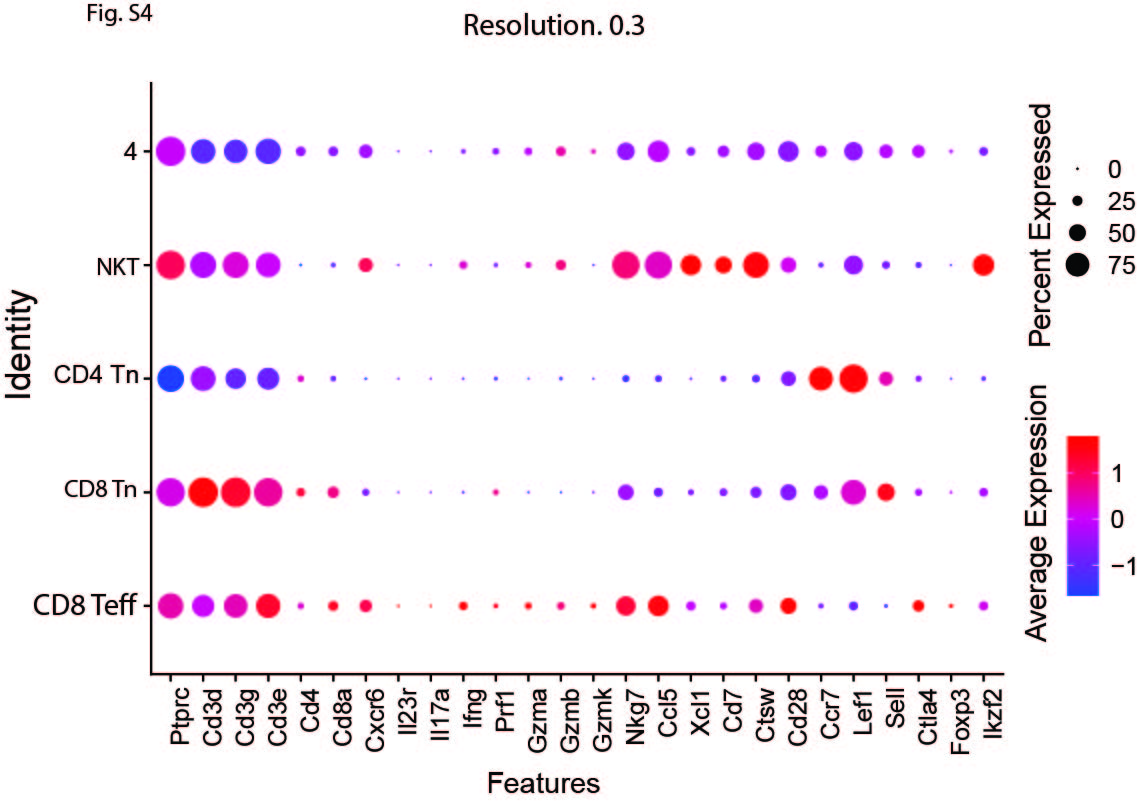

Supplement: Supplementary file 7 [file Image_4.jpg]

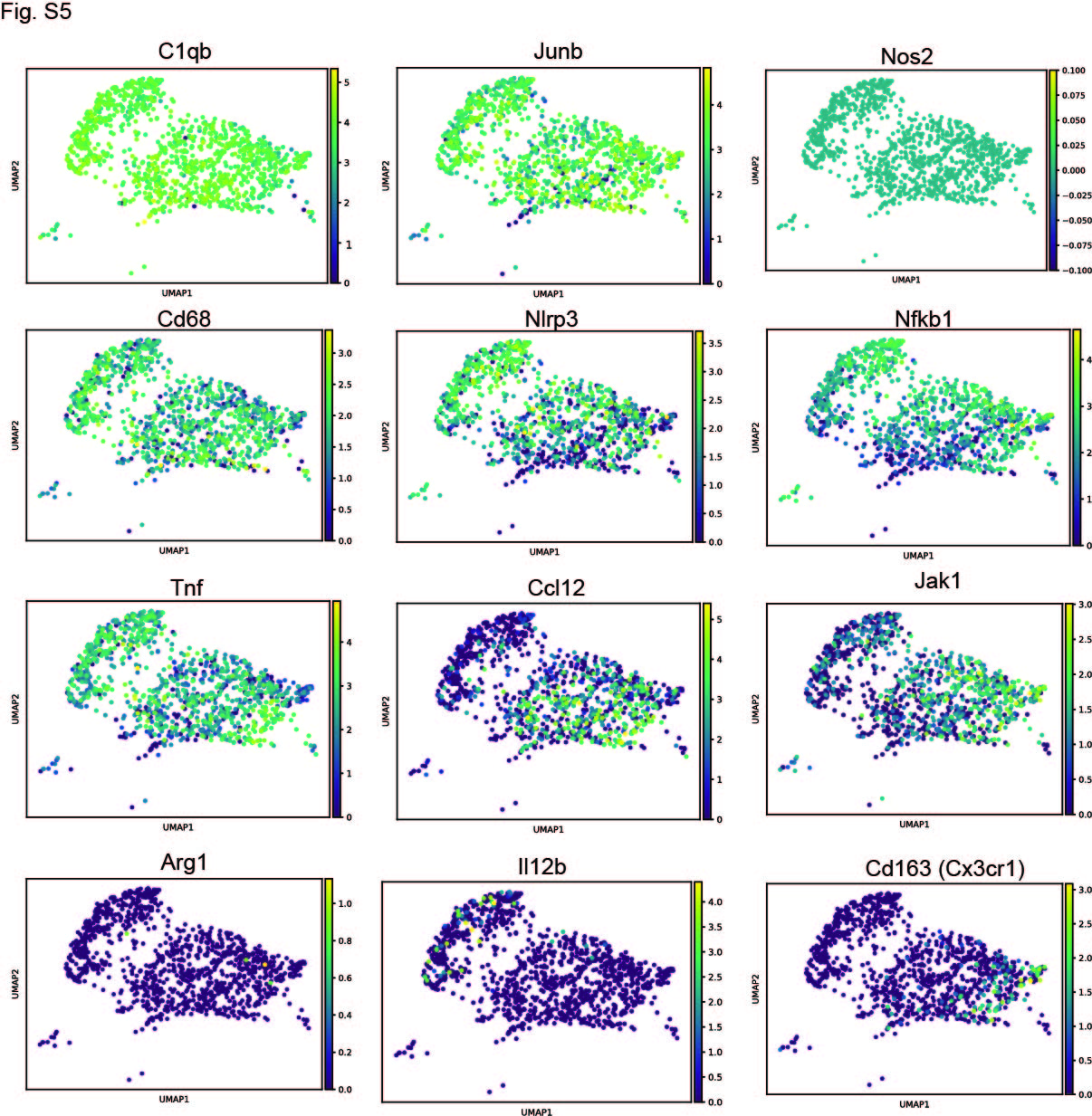

Supplement: Supplementary file 8 [file Image_5.jpg]

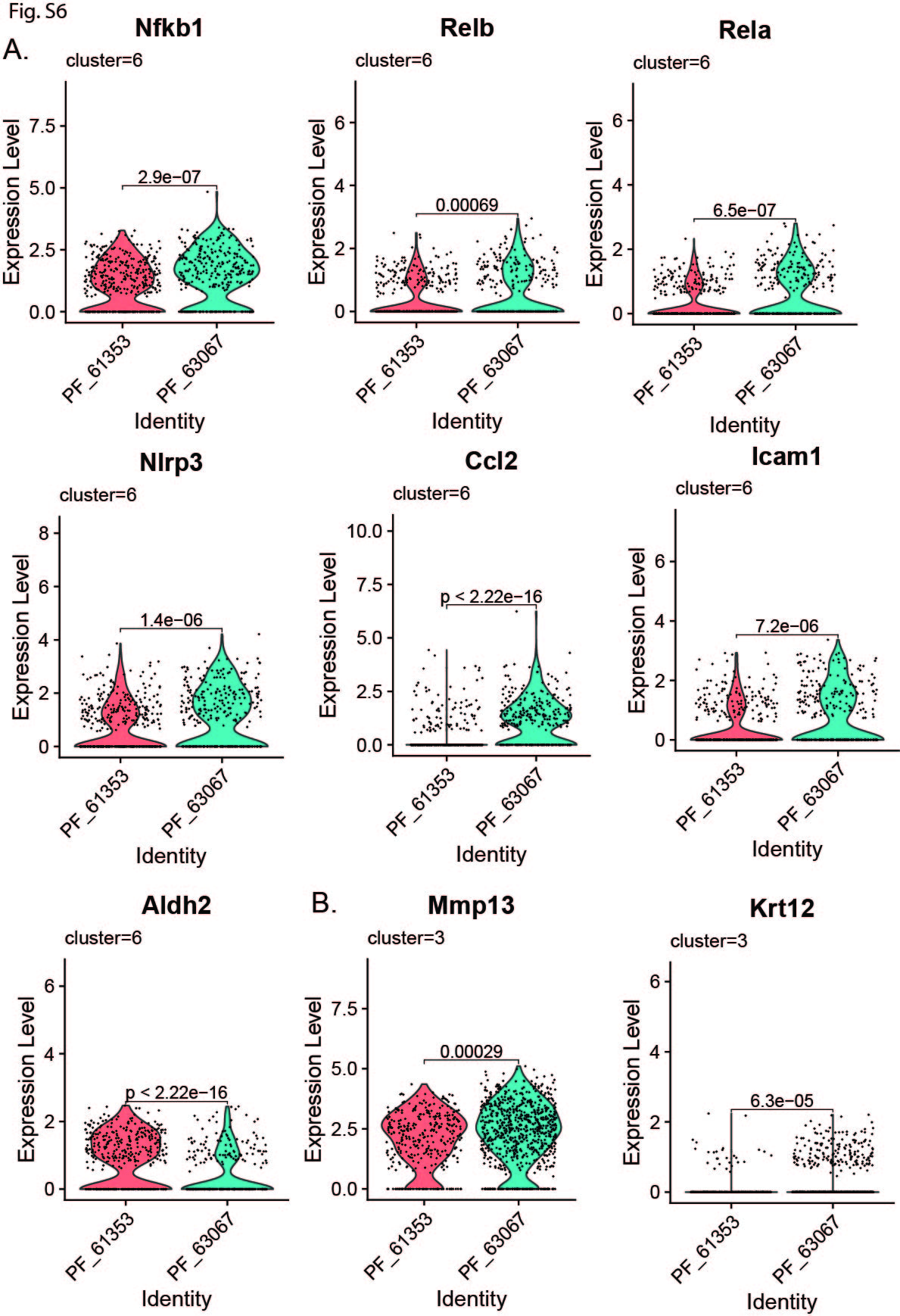

Supplement: Supplementary file 9 [file Image_6.jpg]

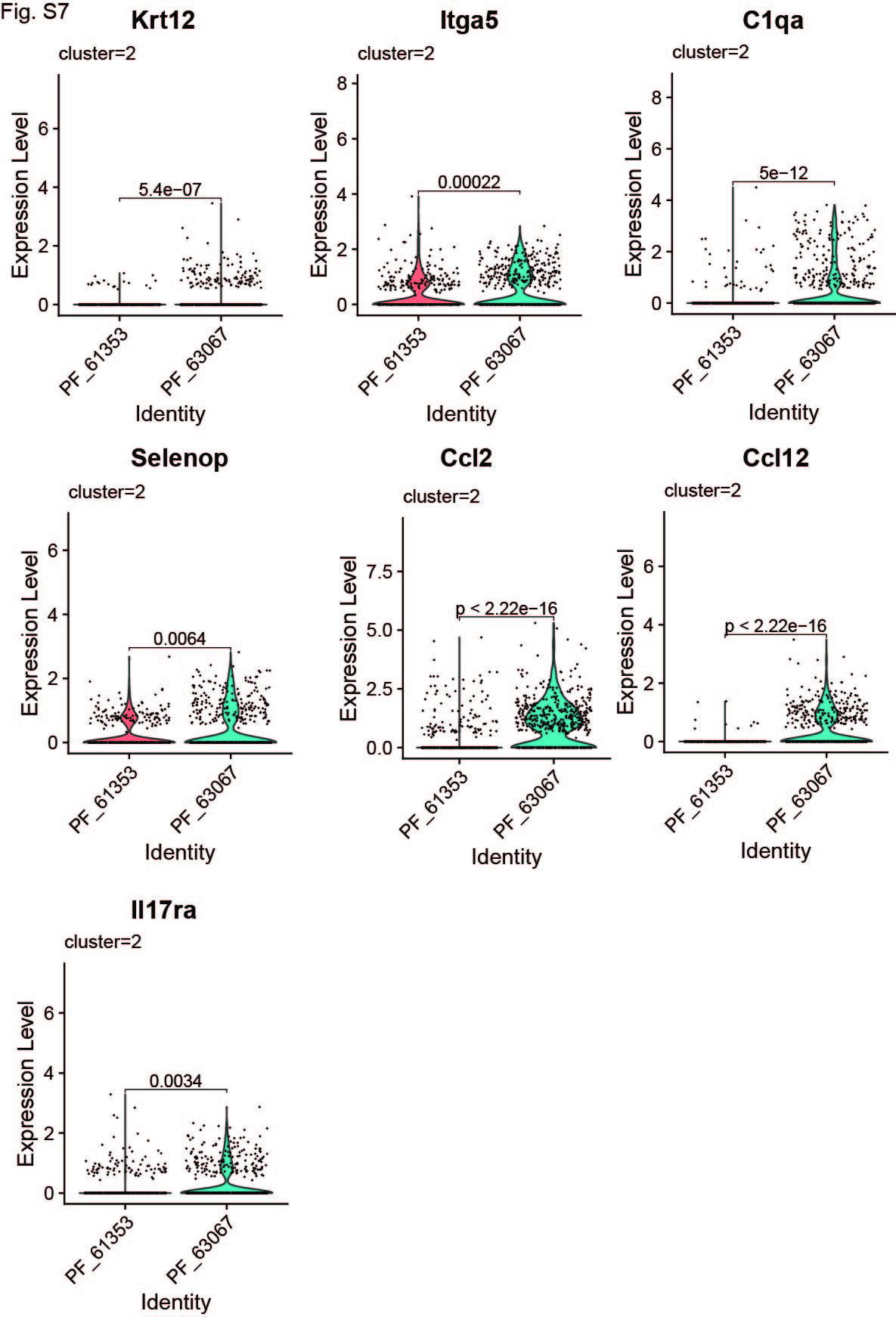

Supplement: Supplementary file 10 [file Image_7.jpg]
